# Rotheln

**Published:** 1883-09-20

**Authors:** 


### *ROTHELN.*

An eruptive disease which nearly answers to this affection as described by authors, is now prevalent in this vicinity, and is proclaimed and treated as *Scarlet fever* by some of our practitioners, which disease it closely resembles.

Rotheln is the German synonim for the *Rhubella* of the English. In America it has not, until within the last few years, been designated by either of the names mentioned. A majority of our practitioners not being conversant with the many later distinctions of the various skin affections, have classed and treated the disease as Scarlet fever, measles, etc.

Rhubella, Rubeola Sine Catarrho, False Measles, German Measles are the several names which have been applied to the disease. It has been defined "A specific eruptive fever, the rash appearing during the first day of the illness, beginning, usually on the face, in rose red spots, extending next day to the body and limbs, subsiding with the fever on the third day, and not preceded by catarrh or followed by desquamation.

It is considered contagious. The diagnosis is often confused by the incidental presence of catarrh or sore throat. It is a mild affection, and usually recovers spontaneously in three or four days. We are in the habit of prescribing a mild laxative in the outset and the use of the following to allay the fever, if high—

Tinct. aconite........................................................................5 drops,
Water.....................................................................................4 ounces.
Teaspoonful every one to two hours.

